# Suppression of LPS-Induced Inflammation and Cell Migration by Azelastine through Inhibition of JNK/NF-κB Pathway in BV2 Microglial Cells

**DOI:** 10.3390/ijms22169061

**Published:** 2021-08-23

**Authors:** Phuong Linh Nguyen, Bich Phuong Bui, Men Thi Hoai Duong, Kyeong Lee, Hee-Chul Ahn, Jungsook Cho

**Affiliations:** College of Pharmacy and Integrated Research Institute for Drug Development, Dongguk University-Seoul, Goyang 10326, Korea; phuonglinh212126@gmail.com (P.L.N.); bichphuong2306@gmail.com (B.P.B.); hoaimenduong@gmail.com (M.T.H.D.); kaylee@dongguk.edu (K.L.)

**Keywords:** azelastine, drug repurposing, c-Jun N-terminal kinase, JNK inhibitor, structure-based virtual screening, neuroinflammation, nuclear factor-kappa B (NF-κB), BV2 microglial cells

## Abstract

The c-Jun N-terminal kinases (JNKs) are implicated in many neuropathological conditions, including neurodegenerative diseases. To explore potential JNK3 inhibitors from the U.S. Food and Drug Administration-approved drug library, we performed structure-based virtual screening and identified azelastine (Aze) as one of the candidates. NMR spectroscopy indicated its direct binding to the ATP-binding site of JNK3, validating our observations. Although the antihistamine effect of Aze is well documented, the involvement of the JNK pathway in its action remains to be elucidated. This study investigated the effects of Aze on lipopolysaccharide (LPS)-induced JNK phosphorylation, pro-inflammatory mediators, and cell migration in BV2 microglial cells. Aze was found to inhibit the LPS-induced phosphorylation of JNK and c-Jun. It also inhibited the LPS-induced production of pro-inflammatory mediators, including interleukin-6, tumor necrosis factor-α, and nitric oxide. Wound healing and transwell migration assays indicated that Aze attenuated LPS-induced BV2 cell migration. Furthermore, Aze inhibited LPS-induced IκB phosphorylation, thereby suppressing nuclear translocation of NF-κB. Collectively, our data demonstrate that Aze exerts anti-inflammatory and anti-migratory effects through inhibition of the JNK/NF-κB pathway in BV2 cells. Based on our findings, Aze may be a potential candidate for drug repurposing to mitigate neuroinflammation in various neurodegenerative disorders, including Alzheimer’s and Parkinson’s diseases.

## 1. Introduction

Neuroinflammation is described as an innate immune response of the cells in the central nervous systems (CNS) to cope with infection and eliminate pathogens, cell debris, and/or misfolded proteins [1,2]. However, in chronic neurological diseases, neuroinflammation becomes persistent and may pose a threat to neuronal cells. This cellular process is known to be mediated by exaggeratedly activated microglia, the main CNS-resident immune surveillance cells [2,3]. In the presence of hazardous stimulations, microglia potentiate inflammation by over-secreting pro-inflammatory molecules such as cytokines, tumor necrosis factor-α (TNF-α), and nitric oxide (NO), eventually contributing to neuronal dysfunction and cell death [4,5]. Moreover, being attracted by the factors released from damaged cells, microglia exhibit enhanced motility toward the sites of injury, which is one of the common features of chronic inflammation during the early phases of neurodegeneration [6]. Accumulating evidence indicates that neuroinflammation is implicated in the principal pathogenesis of a variety of neurodegenerative diseases, including Alzheimer’s disease (AD) and Parkinson’s disease (PD) [7].

c-Jun N-terminal kinases (JNKs) are members of the mitogen-activated protein kinase family and are primarily activated by various stimuli such as environmental stress and pro-inflammatory mediators [8]. Three closely related genes, *JNK1*, *JNK2*, and *JNK3*, are identified to encode 10 splice variants of JNKs [9]. Whereas JNK1 and JNK2 are predominantly expressed in many tissues, JNK3 is almost exclusively found in the brain [10]. Similar to the other organs, JNKs in the brain are expressed in parenchyma cells, connective tissues, and in circulating and resident immune cells such as microglia [11,12,13]. It has been reported that the total amount of nuclear JNKs and the phosphorylation of the transcription factor c-Jun are rapidly and transiently increased in the lipopolysaccharide (LPS)-stimulated microglial cells, leading to augmented levels of inducible NO synthase (iNOS) and pro-inflammatory mediators such as interleukin-6 (IL-6) and NO [12,14,15]. These findings indicate that JNKs play crucial roles in the neuroinflammatory processes underlying various neurodegenerative disorders; thus, JNK inhibitors may offer a promising therapeutic approach for the treatment of these diseases.

In addition to the traditional approaches to discover new drugs, drug repurposing (also referred to as drug repositioning) is a powerful alternative strategy to identify new indications for existing drugs, which takes advantage of saving cost and time [16]. In an attempt to explore potential JNK inhibitors from the U.S. Food and Drug Administration (FDA)-approved drug library, we performed structure-based virtual screening (SBVS) using the 3D structure of JNK3 and obtained a number of compounds with high binding affinity for JNK3. Subsequently, nuclear magnetic resonance (NMR) spectroscopy and in vitro JNK3 kinase assay were employed to validate our findings from SBVS. Among the compounds identified as potential JNK3 inhibitors, azelastine (Aze) was selected for further investigation in this study. Aze is a well-known H1 receptor antagonist with a wide spectrum of anti-allergic and anti-inflammatory activities. It has been demonstrated to inhibit inflammatory mediators, including leukotrienes, kinins, and cytokines. Moreover, it was shown to downregulate the expression of intercellular adhesion molecule-1 and reduce the migration of inflammatory cells [17,18]. Nasal spray and ophthalmic preparations of Aze are commonly indicated to improve symptoms of allergic rhinitis and conjunctivitis, respectively. In some countries, tablet preparation is also available to treat asthma and certain dermatological conditions such as urticaria and pruritus. Although the anti-allergic and anti-inflammatory properties of Aze are documented, the involvement of the JNK pathway in its action remains to be elucidated. Thus, the present study investigated the effects of Aze on LPS-induced phosphorylation of JNK and c-Jun, pro-inflammatory mediators, and cell migration.

Apart from the JNK pathway, microglia-mediated neuroinflammation has also been demonstrated to be controlled by another intracellular cascade activated by nuclear factor-kappa B (NF-κB) [19]. The NF-κB transcription factor regulates the expression of many target genes associated with inflammatory processes [19,20,21]. Thus, inhibition of NF-κB is also beneficial for the treatment of various brain disorders associated with neuroinflammation. We also tested the effect of Aze on LPS-induced nuclear translocation of NF-κB in this study. Notably, LPS initiates neuroinflammatory processes in microglia by releasing various cytokines and eicosanoids [22,23]. Therefore, LPS-treated microglial cells are considered to be a useful cellular model of neuroinflammation to characterize the therapeutic potentials of test compounds [24,25,26]. In this study, we employed LPS-treated BV2 cells, a murine microglial cell line, as a model to investigate the anti-inflammatory and anti-migratory properties of Aze and to elucidate the signaling molecules mediating its effects.

## 2. Results

### 2.1. Structure-Based Virtual Screening

**Figure 1:**
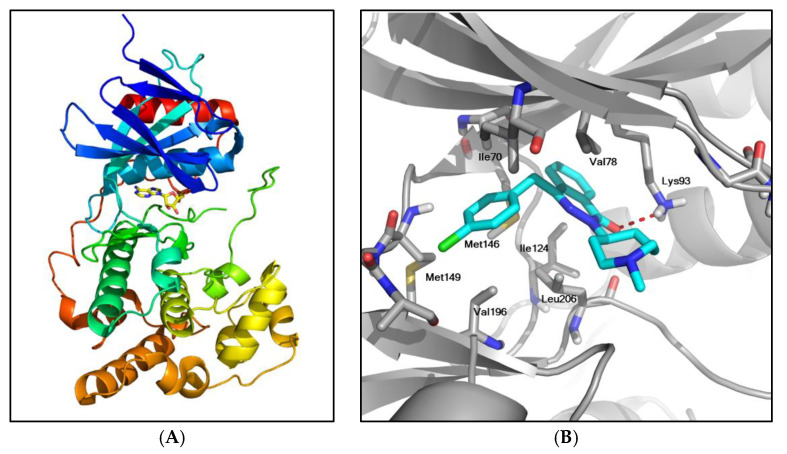
Crystal structure of JNK3 and the structure of JNK3 bound to Aze. (**A**) Overall crystal structure of JNK3 (PDB 4KKE). The bound AMP is displayed as a yellow stick. (**B**) The lowest energy structure of Aze bound to JNK3. JNK3 is displayed as a gray cartoon and Aze as cyan sticks. A hydrogen bond is presented as a red broken line, and the residues in close contact with Aze are highlighted as gray sticks.

The 3D structure of JNK3, retrieved from Protein Data Bank (PDB) (http://www.rcsb.org (accessed on 20 July 2019)), is shown in Figure 1A. The structure of JNK3 is composed of two lobes: a smaller N-terminal lobe with β strands and a larger helical C-terminal lobe. A flexible hinge-like region is connecting the two lobes. The ATP-binding site is located in a deep cleft at the junction of the N and C lobes. Most drug discovery efforts for JNKs have targeted this highly conserved ATP-binding region.

We conducted SBVS with the FDA-approved drug library of 2580 compounds using AutoDock Vina [27] to assess their binding potentials against JNK3. After running the docking experiments, the top-ranked 50 compounds based on the energy scores were selected, including Aze and several anticancer and antiviral agents (data not shown). The changes of Gibbs free energy (ΔG) of their binding to JNK3 were in the range of −11.5~−10.1 kcal/mol, which corresponded to dissociation constants of 3.7~3.9 nM. The ΔG of Aze binding to JNK3 was −10.2 kcal/mol.

The docked pose of Aze to JNK3 with the lowest energy value is shown in Figure 1B. In the proposed structure, Aze is located in the ATP-binding site of JNK3. A hydrogen bond is formed between the oxygen of Aze and the sidechain of Lys93 (the red broken line in Figure 1B). The hydrophobic interactions between Aze and the residues, including Ile70, Val78, Ile124, and Leu206, appear to be important for the formation of the complex. The chlorine of Aze is oriented toward Met149, which plays a pivotal role in the binding of the adenine moiety within the JNK3-ATP complex [28].

### 2.2. Nuclear Magnetic Resonance Spectroscopy

To verify the binding of Aze to JNK3 in the docking simulation, NMR spectroscopy was conducted. Saturation transfer difference (STD) experiment [29] and T_1ρ_- and T_2_-filter experiments [30,31] were performed on Aze with and without JNK3.

**Figure 2:**
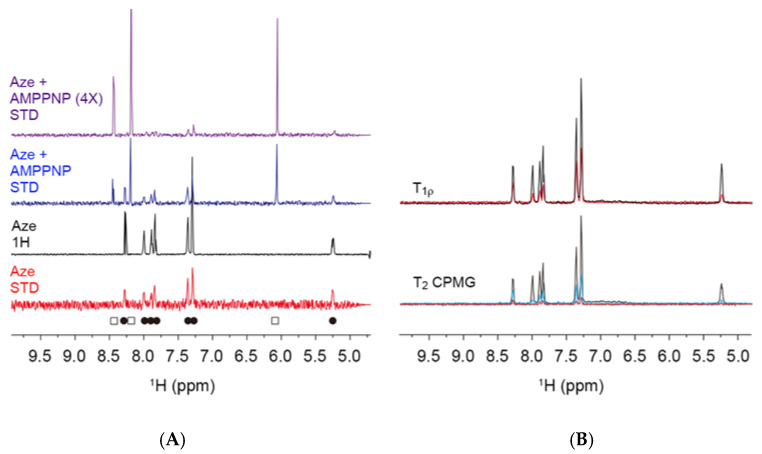
NMR spectroscopy indicating direct binding of Aze to JNK3. (**A**) STD NMR spectrum of Aze in the presence of JNK3 indicates the binding of Aze to JNK3 (red). The 1H NMR spectrum of Aze is shown in black. Aze binds to JNK3 in a competitive manner against AMPPNP (blue and purple). NMR signals from Aze are indicated with filled circles, and those from AMPPNP are presented with open squares at the bottom of the spectra. (**B**) In the T1_ρ_-filter experiments, the spectrum with a 400 ms spin-lock delay (red) shows broader signals than that with a 10 ms delay (black). In the T2-filter experiments, relaxation delays were 8 (black), 80 (blue), and 240 (red), respectively. The broadened 1H signals of Aze in the experiments with longer relaxation delays (400 ms for T1_ρ_ and 240 ms for T2) indicate the direct binding of Aze to JNK3.

STD NMR is a very robust technique to determine whether a small molecule directly binds to a protein. In STD experiments, the resonances of Aze in the presence of JNK3 were detected, indicating direct binding of Aze to JNK3. When AMPPNP, an uncleavable derivative of ATP, was added to JNK3 with Aze, the resonances of both AMPPNP (open square) and Aze (filled circle) were detected. The addition of AMPPNP at 4 molar excess resulted in weakening and almost complete disappearance of the signals of Aze, suggesting that Aze binding to JNK3 occurs at the ATP-binding site and is competitive with AMPPNP (Figure 2A).

Since the relaxation phenomena of a small molecule in the free and bound states are quite different, the T_1ρ_- and T_2_-filter spectra of a compound in the protein-bound status show broadened signals. In Figure 2B, the T_1ρ_ signals of Aze in the presence of JNK3 (black) were sharp with a relaxation delay of 10 ms; however, the signals with a 400 ms delay (red) showed significant line-broadening. Similar results were detected in the T_2_-filter experiments. The spectra with the relaxation delay of 80 (blue) and 240 (red) ms showed more dramatic line-broadening. Thus, the NMR experiments indicate that Aze directly binds to the ATP-binding site of JNK3 in an ATP-competitive manner.

### 2.3. Effect of Aze on JNK3 Activity

After identifying the top-ranked 50 compounds by SBVS, we then evaluated the effects of these compounds on JNK3 activity at the concentration of 10 μM by using in vitro ADP-Glo^TM^ kinase assay. Of these 50 compounds, seven compounds inhibited more than 40% of the JNK3 activity measured in the absence of the test compounds. While SP600125, a well-known JNK inhibitor, inhibited more than 90% of JNK3 activity, Aze inhibited approximately 44% at 10 μM (Figure S1 in the Supplementary Materials).

### 2.4. Effect of Aze on the LPS-Induced Phosphorylation of JNK and c-Jun in BV2 Cells

**Figure 3:**
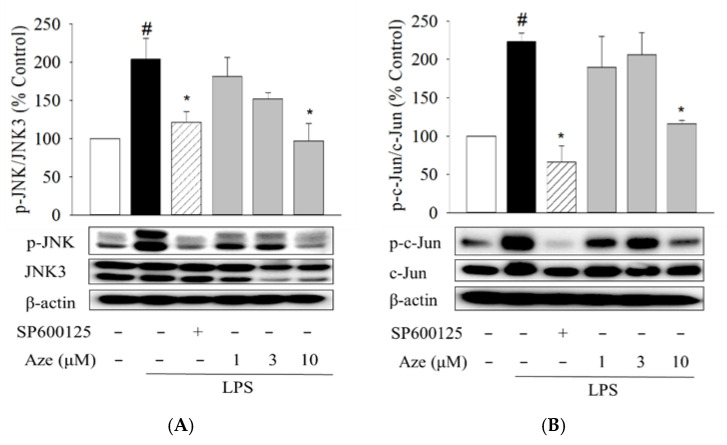
Aze inhibited the LPS-induced phosphorylation of JNK and c-Jun in BV2 cells. Cells were co-treated with LPS (1 μg/mL) and SP600125 (10 μM) or the indicated concentrations of Aze for 24 h. Control cells were treated with vehicle only. Cell lysates were then prepared and analyzed by western blotting using anti-phospho-JNK (p-JNK) and JNK3 antibodies (**A**) or anti-phospho-c-Jun (p-c-Jun) and c-Jun antibodies (**B**). β-Actin was used as a loading control. Representative blots are shown. Data are presented as the mean ± SEM from three independent experiments. # *p* < 0.05 and * *p* < 0.05 vs. vehicle-treated control and LPS-treated cells, respectively.

To validate the docking results, the pharmacological effects of Aze were investigated in parallel with a positive reference compound SP600125 in LPS-treated BV2 cells. We first evaluated the effects of Aze on the LPS-induced phosphorylation of JNK and c-Jun, a major downstream transcription factor of JNK. Our preliminary study revealed that Aze did not exhibit any significant cytotoxicity in BV2 cells at concentrations ranging from 1 to 30 μM (data not shown). Therefore, the effects of Aze were examined within this concentration range throughout the present study. As shown in Figure 3, LPS treatment significantly intensified the phosphorylation of JNK and subsequently augmented the phosphorylation of c-Jun in BV2 cells. The levels of phosphorylated JNK and c-Jun were markedly inhibited by Aze at the concentration of 10 μM. The degrees of inhibition were comparable to those shown by SP600125.

### 2.5. Effect of Aze on the LPS-Induced Production of Pro-Inflammatory Mediators in BV2 Cells

**Figure 4:**
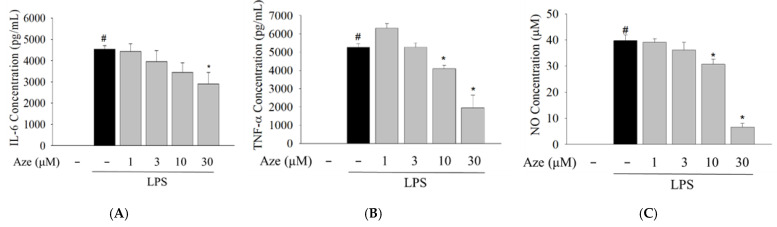
Aze suppressed the LPS-stimulated production of pro-inflammatory molecules in BV2 cells. Cells were treated with LPS (1 μg/mL) and the indicated concentrations of Aze. Control cells were treated with vehicle only. Culture media were collected after 24 h of treatment, and the levels of IL-6 (**A**), TNF-α (**B**), and NO (**C**) were determined as described in the Materials and methods. Data are presented as the mean ± SEM from three independent experiments. # *p* < 0.05 and * *p* < 0.05 vs. vehicle-treated control and LPS-treated cells, respectively.

The critical role of the JNK pathway in the production of pro-inflammatory molecules has been elucidated in numerous studies [32,33,34]. Using ELISA and Griess assays, we next explored whether Aze exerted any effect on the production of LPS-induced pro-inflammatory mediators in BV2 cells. In parallel with our previous findings [34,35], LPS treatment drastically enhanced the levels of pro-inflammatory factors, including IL-6, TNF-α, and NO (Figure 4). Simultaneous treatment with LPS and Aze significantly and concentration-dependently inhibited the LPS-stimulated production of these mediators (Figure 4).

### 2.6. Effect of Aze on the LPS-Induced Expression of COX2 and iNOS in BV2 Cells

**Figure 5:**
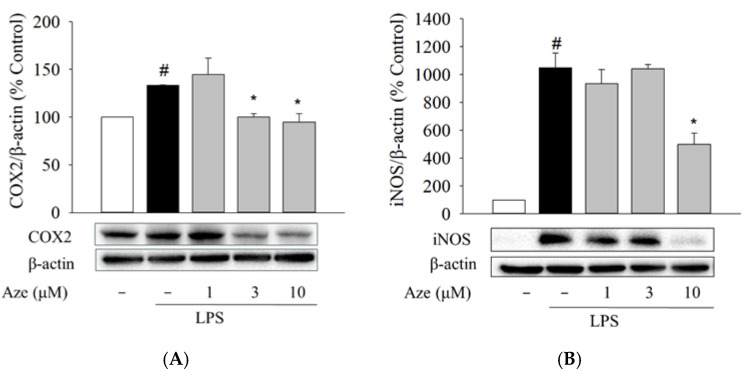
Aze reduced the LPS-stimulated protein levels of COX2 and iNOS in BV2 cells. Cells were co-treated with LPS (1 μg/mL), and the indicated concentrations of Aze for 24 h. Control cells were treated with vehicle alone. The expressions of COX2 (**A**) and iNOS (**B**) were determined by Western blotting analysis, as described in the Materials and Methods. Representative blots are shown. Data are presented as the mean ± SEM from three independent experiments. # *p* < 0.05 and * *p* < 0.05 vs. vehicle-treated control and LPS-treated cells, respectively.

COX2 and iNOS are the two critical enzymes involved in the production of pro-inflammatory mediators by LPS stimulation in microglia [36]. We next determined whether the suppression of pro-inflammatory mediators by Aze is correlated with the regulation of COX2 and iNOS expression in BV2 cells. Western blotting analyses revealed that the enhancement of COX2 and iNOS expression by LPS treatment was significantly attenuated by Aze (Figure 5).

### 2.7. Effect of Aze on the LPS-Induced Cell Migration in BV2 Cells

**Figure 6:**
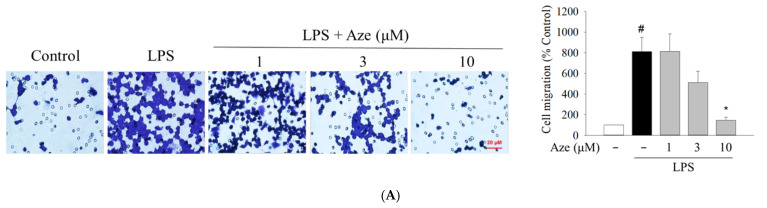

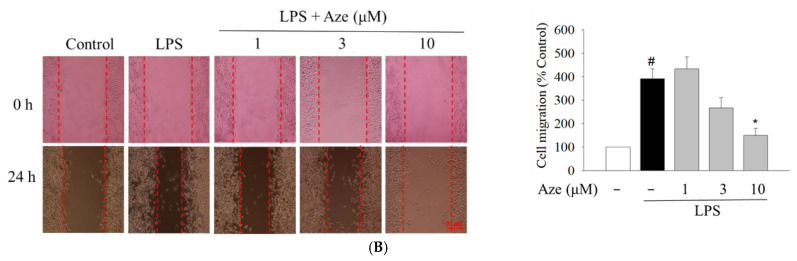
Aze diminished the LPS-stimulated cell migration in BV2 cells. Cells were co-treated with LPS (1 μg/mL) and a series of concentrations of Aze for 24 h. Control cells were treated with vehicle alone. The alterations in cell migration were assessed by transwell (**A**) and wound healing (**B**) assays, as described in the Materials and methods. Representative microscopic images are shown (scale bar, 20 μm). Data are presented as the mean ± SEM from three independent experiments. # *p* < 0.05 and * *p* < 0.05 vs. vehicle-treated control and LPS-treated cells, respectively.

Apart from the production of pro-inflammatory mediators, the migration of microglial cells toward the infected sites in response to inflammatory stimuli also plays an essential role in the propagation of neuroinflammation [6,37]. Therefore, we examined whether Aze exerted any impact on the LPS-induced migration of BV2 cells. By performing transwell (Figure 6A) and wound healing assays (Figure 6B), we observed that the cell migration enhanced by LPS treatment was dramatically diminished by Aze.

### 2.8. Effect of Aze on the LPS-Induced Nuclear Translocation of NF-κB in BV2 Cells

**Figure 7:**
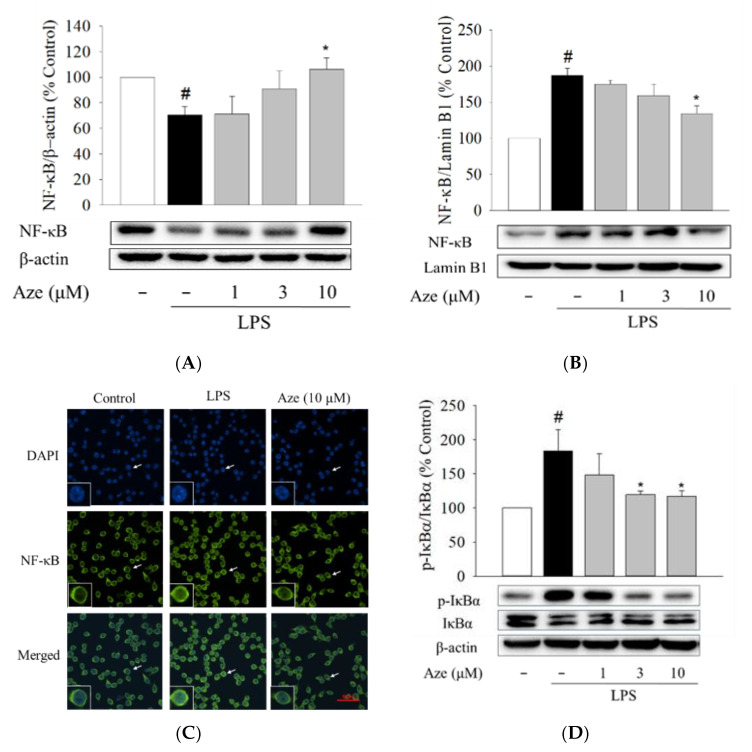
Aze suppressed the LPS-induced nuclear translocation of NF-κB by inhibiting the phosphorylation of IκBα. BV2 cells were treated with various concentrations of Aze in the presence of LPS (1 μg/mL) for 24 h. Control cells were treated with vehicle alone. Protein expression levels for cytosolic NF-κB (**A**) and nuclear NF-κB (**B**) were evaluated by western blotting using anti-NF-κB p65 antibodies. β-Actin and lamin B1 were used as loading controls for the cytosolic and nuclear fractions, respectively. Representative blots are shown. Data are presented as the mean ± SEM from at least three independent experiments. Immunofluorescence images showing inhibition of NF-κB nuclear translocation by Aze (**C**). Cells were co-treated with LPS (1 µg/mL) and Aze (10 µM) for 24 h, and immunocytochemical analysis was carried out using DAPI and anti-NF-κB p65 antibodies, as described in the Materials and Methods. Representative images are shown. The arrow in each image indicates the magnified cell shown in the inset. Scale bar, 50 µm. Western blotting analyses were conducted using anti-phospho-IκB and anti-IκB antibodies (**D**). Cells were co-treated with LPS (1 µg/mL) and a series of concentrations of Aze for 24 h. β-Actin was used as an internal control. Data are presented as the mean ± SEM from at least three independent experiments. # *p* < 0.05 and * *p* < 0.05 vs. vehicle-treated control and LPS-treated cells, respectively.

The nuclear factor-kappa B (NF-κB) is a key signaling molecule that regulates the levels of pro-inflammatory factors following LPS treatment [19]. Thus, we then examined the involvement of the nuclear translocation of the NF-κB p65 subunit in the anti-inflammatory activities of Aze. In comparison with the vehicle treatment, LPS treatment significantly increased the level of nuclear NF-κB, while it decreased the cytosolic NF-κB level (Figure 7), inducing translocation of NF-κB to the nucleus. Aze exerted a notable inhibition of the LPS-stimulated nuclear translocation of NF-κB, with a simultaneous increase in the cytosolic NF-κB level (Figure 7). To verify these findings, we further performed immunocytochemical analysis. As shown in Figure 7C, the NF-κB p65 subunit was primarily localized in the cytoplasm, which was then switched to the nucleus after exposure to LPS. Aze, at 10 µM, was found to suppress the LPS-induced nuclear translocation of NF-κB.

It is well documented that the nuclear translocation of NF-κB is triggered by the phosphorylation of IκBα [38]. Therefore, we also investigated the effect of Aze on the phosphorylation of IκBα. Immunoblotting analyses revealed that the phosphorylation of IκBα was significantly increased by LPS in BV2 cells, which was significantly reversed by Aze at concentrations of 3 μM and above. These data indicate that Aze inhibits the LPS-induced nuclear translocation of NF-κB through inhibition of IκBα phosphorylation. Taken together, these findings indicate that Aze exerts anti-inflammatory and anti-migratory effects through inhibition of JNK phosphorylation, which subsequently leads to the inhibition of IκBα phosphorylation, resulting in inhibition of NF-κB nuclear translocation.

## 3. Discussion

The JNKs, depending on cellular alterations, are involved in the regulatory mechanisms of many physiological processes such as brain development, repair, and memory function. In addition, JNKs are also important modulators in inflammation and stress responses, which are critical features of the etiology and progression of many neurodegenerative diseases such as AD, PD, and multiple sclerosis [32,39,40]. In particular, JNK3, one of the three highly homologous JNKs, is abundantly and almost exclusively expressed in the brain and functions as an essential player of the neuronal immune response in various neuropathological conditions [13,41]. For example, elevated expressions of JNK3, c-Jun, as well as levels of pro-inflammatory cytokines, including TNF-α, IL-6, and IL-1β, were observed in the hippocampus of the chronic social defeat stress mice model [42]. Moreover, in rats subjected to transient middle cerebral artery occlusion, several JNK3 inhibitors are shown to restore anti-oxidant enzyme activity and reduce the levels of oxidative stress-induced phosphorylation of JNK and neuroinflammatory mediators [43]. These reports strongly suggest that inhibition of JNK3 can be a favorable strategy to alleviate the progression of neuroinflammation and neurodegeneration. In our study using SBVS, Aze, an antihistamine drug acting at the histamine H1 receptor, was found to be a potential JNK3 inhibitor. We further demonstrated in the present study that Aze exerted anti-inflammatory and anti-migratory effects through inhibition of the JNK/NF-κB pathway in BV2 cells.

Drug repurposing is the process of establishing novel therapeutic potentials of pre-existing drugs. It has gained substantial attention as an alternative approach in drug discovery and development since it is a time- and cost-effective strategy to characterize additional pharmacological actions and to identify new therapeutic indications [16,44]. In the context of drug repurposing, we employed computational docking techniques to explore potential JNK3 inhibitors from commercially available approved drugs [45]. In the molecular docking study, interactions between the desired target and a given drug are determined based on the structural input of the drug and target [46]. We carried out SBVS of the FDA-approved drug library of 2580 compounds using AutoDock Vina to identify potential JNK3 inhibitors. The top-ranked 50 compounds, which showed high binding affinities with one-digit nanomolar concentrations of the dissociation constants, were selected by virtual screening. Aze was one of these compounds with a docking score of −10.2 kcal/mol. Using the 3D structure of JNK3, the docked pose of Aze to JNK3 with the lowest energy value was also elucidated (Figure 1). The binding mode of Aze to JNK3 was proposed based on our findings from the docking simulation. Aze forms a hydrogen bond with the sidechain of Lys93 and shows close contact with Met149, which is a very important residue in the binding of the adenine moiety of ATP. Several hydrophobic residues, such as Ile70, Val78, Ile124, Val196, and Leu206, wrap Aze to stabilize the protein-drug complex via hydrophobic interactions (Figure 1).

To further verify whether Aze directly binds to JNK3, NMR experiments were performed. NMR spectroscopy is a very powerful tool to investigate protein-ligand interactions [47]. The NMR phenomena of a small molecule in the protein-bound and -unbound statuses are quite different. For example, a small molecule tumbles fast and has long T_1_ and T_2_ relaxation times in a free status; however, it behaves like a large molecule or a part of the large molecule in a bound status. Thus, the T_1_ and T_2_ relaxation times of a small molecule in the protein-bound status are shorter than those in the free status. With longer relaxation delays, e.g., 400 ms for T_1ρ_ or 240 ms for T_2_ experiments, the NMR signals of Aze were significantly broadened (Figure 2B), indicating that the compound was bound to JNK3. STD NMR is also a robust technique to discriminate protein binders. Saturation on a protein (on-resonance) gives rise to spin diffusion toward the bound small molecule. However, the off-resonance saturation cannot induce spin diffusion; thus, the difference between the off-resonance and on-resonance spectra can be used to detect the signals of the protein-bound small molecule. In our NMR experiments with Aze and JNK3, we detected both longitudinal (T_1_) and transverse (T_2_) fast relaxation and the STD signal of Aze in the presence of JNK3 (Figure 2). Moreover, the STD NMR experiments with Aze in the presence of JNK3 and AMPPNP showed competition of the two small molecules on the same site of the protein, confirming that Aze binds to the ATP-binding site of JNK3. Our observation strongly supports that SBVS is a valuable approach to discover protein binders in drug repurposing. Collectively, these results validate the SBVS data, demonstrating the high-affinity binding of Aze to JNK3 at the ATP-binding site. On the basis of these favorable results, we continued to examine the effects of Aze on the phosphorylation of JNK and c-Jun using BV2 microglial cells as a cellular model.

Microglia are the first line of immunological defense against various infectious and injurious agents that breach the CNS [3,48]. Microglia activated by various insults and genetic and environmental factors can stimulate and exacerbate inflammatory responses within the CNS, causing neuroinflammation and neurodegeneration [21,48]. For instance, LPS, an endotoxin found in the outer membrane of Gram-negative bacteria, induces a systemic inflammatory response by binding to the Toll-like 4 receptor on the microglia surface, which in turn activates several transduction pathways such as JNK and NF-κB [34,35]. LPS-treated BV2 microglial cells are widely employed to investigate the cellular and molecular pathways related to neuroinflammation [34,35,49]. Moreover, it is well-established that mRNAs for JNKs and their corresponding proteins are expressed in the cultured microglial cells [50]. Therefore, the present study used LPS-stimulated BV2 cells to investigate the effects of Aze on JNK3 activity by using western blotting analysis. In agreement with previous findings [34], our study revealed that, following LPS treatment, JNK3 was phosphorylated and activated in BV2 cells. The LPS-induced JNK3 phosphorylation was effectively suppressed by Aze (Figure 3). The activation of JNKs is known to persist with N-terminal phosphorylation of a transcript factor, c-Jun, ultimately regulating inflammation responses [51]. We also found that Aze subsequently inhibited the LPS-induced phosphorylation of c-Jun. These results demonstrate that Aze inhibits the LPS-induced phosphorylation of JNK3 and c-Jun in BV2 cells, validating our in silico and NMR data.

Aze is an antihistamine clinically available as an anti-allergic drug and used for relieving nasal symptoms [52]. Therefore, the physicochemical properties of Aze and its pharmacokinetic and safety profiles are well documented. In an effort to elucidate additional pharmacological actions of Aze, we continued to examine its effects on inflammatory mediators and cell migration in LPS-treated BV2 cells. Neuroinflammation results primarily from the activation of microglia, which can secrete inflammatory mediators such as IL-6 and TNF-α or toxic substances such as NO. In the present study, the levels of IL-6, TNF-α, and NO markedly increased in the presence of LPS, similar to previous reports [34,35]. However, Aze reversed the LPS-induced enhancements of these mediators (Figure 4). The elevated level of COX2 is observed in the process of neuronal damage and neuroinflammation, which is primarily responsible for the diffusion of various prostaglandins [53]. In addition, high levels of NO are continuously synthesized by the inducible isoform of NOS (iNOS) under pathological conditions or after exposure to neurotoxic agents [54]. Thus, the sustained up-regulation of these two enzymes contributes to the progressive damage, exacerbating the characteristics of neurodegenerative diseases. We observed that Aze significantly alleviated the LPS-induced protein levels of COX2 and iNOS (Figure 5). These findings demonstrate the anti-inflammatory effect of Aze through inhibition of JNK3 and c-Jun in BV2 cells.

In many pathological conditions in the CNS, the activated microglia autonomously converge onto the injured sites and release various inflammatory molecules, which causes worsening of inflammation and promotes neurodegeneration [55,56]. Moreover, several studies have demonstrated that the messenger molecule NO produced in the lesion sites plays a crucial role as a chemoattractant and regulates cell migration [57,58,59]. Altogether, it is concluded that microglia exhibit a highly motile characteristic to mediate the rapid extension of chronic inflammation caused by exaggeratedly activated microglia. Therefore, inhibition of cell migration is an important strategy to suppress inflammatory responses. Our previous reports ascertained that LPS treatment increased BV2 cell migration [34,35]. Subsequently, using transwell and wound healing assays in this study, we showed that the LPS-induced BV2 cell migration was markedly suppressed by Aze (Figure 6).

NF-κB is a transcription factor that mediates the expression of many genes associated with innate and adaptive immune responses as well as inflammation [60]. In the resting state, it is retained in the cytoplasm as a heterodimer complexed with its inhibitory protein IκBα. As a consequence of the intracellular kinase signaling cascades, IκBα is phosphorylated by a ternary IκB kinase (IKK) complex, eventually resulting in ubiquitination and degradation of IκBα. Thus, the interaction of IκBα with NF-κB is disrupted, and NF-κB is liberated to translocate to the nucleus, where it can bind to specific promoter components to regulate the expression of specific cellular genes [19,21,60]. To further understand the intracellular signaling pathways associated with the inhibition of pro-inflammatory molecules and cell migration, we then evaluated the effect of Aze on the NF-κB pathway. Consistent with our previous studies [34,35], LPS induced phosphorylation of IκBα, subsequently leading to nuclear translocation of NF-κB. Aze significantly inhibited the LPS-induced IκBα phosphorylation and thereby inhibited nuclear translocation of NF-κB (Figure 7). These findings suggest that the anti-neuroinflammatory effect of Aze may also be attributed to its inhibition of the NF-κB pathway. Interestingly and expectedly, SP600125 has also been shown to remarkably attenuate the LPS-triggered IκBα phosphorylation as well as nuclear translocation of NF-κB in BV2 cells [34,61].

**Figure 8:**
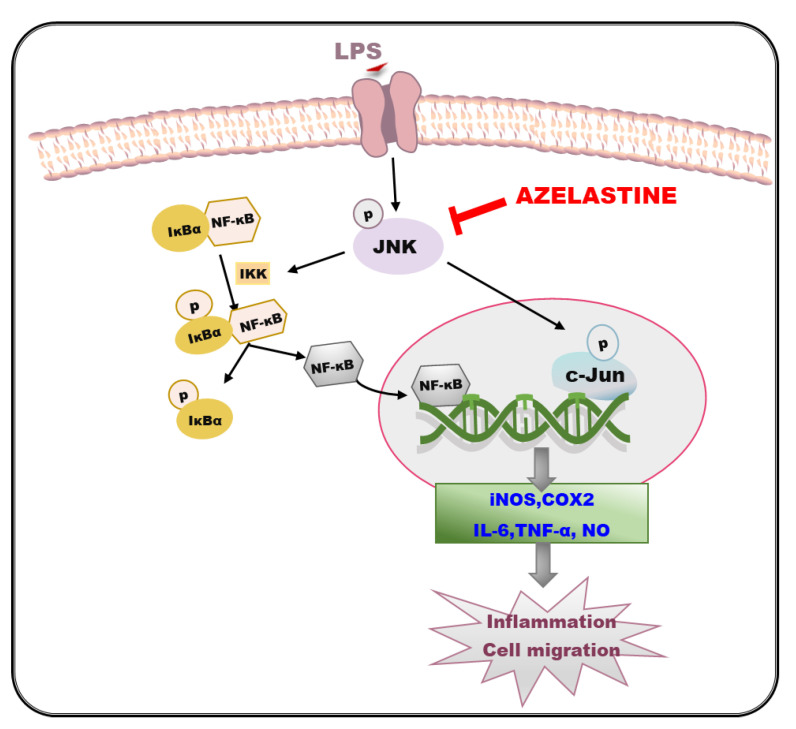
Schematic diagram illustrating the cellular mechanisms involved in the anti-inflammatory and anti-migratory effects of Aze in LPS-treated BV2 cells. IKK, IκB kinase; IκBα, inhibitory kappa Bα; NF-κB, nuclear factor-kappa B; iNOS, inducible nitric oxide synthase; COX2, cyclooxygenase-2; IL-6, interleukin-6, TNF-α, tumor necrosis factor-α; NO, nitric oxide.

Taken together, our findings demonstrate in this study that Aze suppresses the LPS-induced production of pro-inflammatory mediators and cell migration through inhibition of JNK3, which serves as an upstream signal to subsequently inhibit NF-κB nuclear translocation. The JNK/NF-κB pathway inhibited by Aze in BV2 cells is summarized in a schematic diagram (Figure 8). This is the first report elucidating JNK/NF-κB-mediated anti-inflammatory and anti-migratory effects of Aze. Based on our findings, Aze may be a potential candidate for drug repurposing to alleviate the neuroinflammation associated with various neurodegenerative disorders, including AD and PD. Although BV2 cells share the main functional characteristics with microglia of human origin to become a reliable cellular model for studies on microglia-associated neurodegenerative diseases [62], it would be worth confirming and validating our findings in human microglia cells to provide better relevance for human neurodegenerative diseases.

## 4. Materials and Methods

### 4.1. Materials and Chemicals

Aze was purchased from Selleckchem (Houston, TX, USA). SP600125 was obtained from Calbiochem (Darmstadt, Germany). LPS (*Escherichia coli*, serotype 011:B4), mouse anti-β-actin (1:2000, cat# A5316) and rabbit anti-lamin B1 (1:2000, cat# SAB1306342) antibodies, dimethyl sulfoxide (DMSO), and adenylyl-imidodiphosphate (AMPPNP) were obtained from Sigma-Aldrich (St. Louis, MO, USA). Alexa Fluor 488-conjugated anti-mouse immunoglobulin G (IgG) and 4′,6-diamidino-2-phenylindole dihydrochloride (DAPI) were obtained from Thermo Scientific (Rockford, IL, USA). Antibiotic-antimycotic reagent, Dulbecco’s modified Eagle medium (DMEM), and fetal bovine serum (FBS) were purchased from Gibco BRL (Grand Island, NY, USA). The rabbit anti-phospho-IκBα (1:1000, cat# ab97783) antibody was supplied by Abcam (Cambridge, MA, USA). Rabbit anti-phospho-JNK (1:1000, cat# 4668), rabbit anti-JNK3 (1:1000, cat# 2305), rabbit anti-phospho-c-Jun (1:1000, cat# 9261), rabbit anti-c-Jun (1:1000, cat# 9165), rabbit anti-iNOS (1:1000, cat# 2982), rabbit anti-COX2 (1:1000, cat# 4842), mouse anti-NF-κB p65 (1:1000, cat# 6956), mouse anti-IκBα (1:1000, cat# 4814) antibodies, and horseradish peroxidase-conjugated anti-rabbit (1:2000, cat# 7074) and anti-mouse IgG (1:2000, cat# 7076) were purchased from Cell Signaling Technology (Danvers, MA, USA).

### 4.2. Virtual Screening

The 3D X-ray crystallographic structure of JNK3 in complex with an inhibitor (PDB: 4KKH) with a resolution of 2.0 Å was used in this study. It contains 363 residues of JNK3 spanning from Ser40 to Glu402. Prior to defining the docking site, the JNK3 structure was prepared by adding polar hydrogens and Gasteiger charges and removing water molecules and ligands. The structure data file (SDF) of the FDA-approved drug library consisting of 2580 compounds (structure data file: SDF) was downloaded from the website http://www.selleckchem.com (accessed on 20 July 2019) and used for this study. First, all compounds were prepared by the addition of hydrogen atoms to the structures and transformed into a valid 3D conformer by using the Open Babel Package, version 2.4.0 (http://openbabel.org) (accessed on 20 July 2019) [63]. Later, the torsion bonds were automatically set for the ligand library.

AutoDock Vina was used for the molecular docking study. The docking site was set as rigid at the ATP-binding site, including the residues Ile70, Gln75, Leu144, Met146, Met149, Asn194, and Asn152. The grid box, with dimensions of 40 × 40 × 40 Å, encompassed all residues of the active site. The parameters of the search algorithm were kept at the default values of the software. The compounds were docked into the defined ATP-binding site. During the docking process, docking simulations were performed by employing the Lamarckian genetic algorithm, and the receptor was kept rigid, while the ligands were kept flexible to rotate and explore the most probable binding conformations. After the docking process, the scores of the binding free energy were generated. The compounds in the library were sorted by docking score, and the top 50 compounds were selected for further experiments. Pymol (The PyMOL Molecular Graphics System, Version 2.1.0 Schrödinger, LLC, New York, NY, USA) was used for visualization of the docked results.

### 4.3. NMR Study for Binding of Aze to JNK3

For the NMR experiments, the recombinant catalytic domain of JNK3 was prepared as described previously [64]. The protein concentration in all NMR experiments was 10 μM, and that of Aze was 200 μM. All NMR data were measured at 25 °C on a Bruker Avance II 800 MHz NMR spectrometer equipped with a cryogenic probe at Korea Basic Science Institute. T_1ρ_ experiments were recorded with the spin-lock relaxation delay of 10 and 400 ms, and T_2_ experiments used the Carr-Purcell-Meiboom-Gill (CPMG) sequence with the delay of 8, 80, 160, and 240 ms. For STD NMR, the reference experiments were acquired with the off-resonance saturation at 30 ppm, and the saturation experiments were acquired with the on-resonance saturation at 0.5 ppm. The duration of the saturation was 3 s for both on- and off-resonance experiments. For the competition STD experiments of Aze and AMPPNP to JNK3, the concentration of Aze was 200 μM, and those of AMPPNP were 200 or 800 μM. The sweep widths were 11,160.7 Hz, and the points of time-domain were 32 K for all experiments. All data were processed and analyzed with the software Mnova (Mestrelab Research, Santiago de Compostela, Spain).

### 4.4. JNK3 Activity Assay

The inhibitory effects of the selected compounds on JNK3 activity were evaluated by the JNK3 ADP-Glo^TM^ assay (cat# V6930, Promega, Madison, WI, USA), according to the manufacturer’s instructions. Briefly, the reaction was prepared with a final concentration of 25 ng of active JNK3, 50 μM ATP, 0.5 μg of p38 peptide substrate, 1× reaction buffer, and 10 μM of the test compounds. The experiment was set up with four controls, namely, without ATP, without active JNK3, without test compound (negative control), and with SP600125 (positive control). The JNK3 solution was pre-incubated with each compound for 10 min at room temperature. The p38 peptide substrate and ATP were added to make the final concentrations of 0.5 μg and 50 μM, respectively, to start the kinase reaction. The mixture was incubated for 1 h at room temperature. An equal volume of ADP-Glo™ Reagent was added to terminate the kinase reaction and deplete the remaining ATP. The kinase detection reagent was added to simultaneously convert ADP to ATP, and the newly synthesized ATP was measured using a luciferase/luciferin reaction. The measured luminescence was converted to ADP concentrations by using an ATP-to-ADP conversion curve.

### 4.5. BV2 Cell Culture and Treatment of Cells

BV2 microglial cells were maintained in DMEM containing 10% heat-inactivated FBS and 1% antibiotic-antimitotic agents at 37 °C in a humidified incubator with 95% O_2_ and 5% CO_2_ as previously described [35]. Aze was dissolved in DMSO to a stock solution of 10 mM and subsequently passed through a 0.22 μm sterile filter. Cells were treated with LPS (1 μg/mL) and/or a series of concentrations of Aze for 24 h. Cells exposed to serum-free media containing 0.3% DMSO were used as the vehicle-treated control groups.

### 4.6. Western Blotting

BV2 cells were plated on 35 mm culture dishes at a density of 1 × 10^6^ cells/dish and treated with different concentrations of Aze for 24 h with or without LPS (1 µg/mL). After the desired treatment, cells were lysed in the lysis buffer, as described previously [34,35]. To evaluate the effect of Aze on the nuclear translocation of NF-κB, BV2 cells were plated on 60 mm dishes at a density of 2.5 × 10^6^ cells/dish and co-treated with LPS and the indicated concentrations of Aze. After the desired treatment, cytosolic and nuclear fractions were prepared using NE-PER Nuclear and Cytoplasmic Extraction Reagents (cat# 78835, Thermo Fisher, Rockford, IL, USA), according to the manufacturer’s instructions. The lysates and the cytosolic and nuclear fractions were stored at −80 °C until use. The protein concentrations were determined using a Bio-Rad DC protein assay kit (Bio-Rad, Hercules, CA, USA).

Immunoblotting experiments were conducted as reported previously [34,35]. Briefly, equal amounts of proteins were separated by SDS-polyacrylamide gel electrophoresis and transferred onto nitrocellulose membranes (Merck Millipore Ltd., Billerica, MA, USA). To block nonspecific bindings, the membranes were incubated with Tris-buffered saline with 0.1% Tween 20 (TBS-T) containing 5% (*w*/*v*) skim milk (BD Bio-sciences, San Jose, CA, USA) for 1 h at room temperature. Then, the membranes were incubated at 4 °C overnight with targeted primary antibodies in 5% bovine serum albumin, followed by incubation with HRP-conjugated secondary antibodies for 2 h. Finally, the blots were scanned by a Bio-Rad ChemiDoc XRS imaging system using enhanced chemiluminescence reagents (Bio-Rad, Hercules, CA, USA).

### 4.7. Determinations of IL-6, TNF-α, and NO

To evaluate the effects of Aze on the LPS-induced production of pro-inflammatory mediators, the levels of IL-6 and TNF-α were determined using enzyme-linked immunosorbent assays (ELISAs), while NO concentration was measured by the Griess reaction, as reported previously [34,35]. Overall, BV2 cells seeded onto 24-well plates at a density of 2.5 × 10^5^ cells/well were treated for 24 h with LPS (1 µg/mL) in the absence or presence of Aze at the indicated concentrations. After treatment, the conditioned media were cautiously collected and centrifuged at 1500 rpm at 4 °C for 10 min. The supernatants were then stored at −80 °C until use. Mouse IL-6 and TNF-α ELISA kits (cat# K0331230 and K0331186, respectively, KomaBiotech, Seoul, Korea) and Griess reagent (cat# G2930, Promega Corporation, Madison, WI, USA) were used following the manufacturers’ recommendations. The absorbance was read at 450 nm on a microplate reader (SpectraMax M2e, Molecular Devices, Sunnyvale, CA, USA). The concentrations of IL-6, TNF-α, and NO were calculated from the respective standard curves generated simultaneously.

### 4.8. Cell Migration Assays

#### 4.8.1. Transwell Migration Assay

BV2 cells were plated at a density of 1 × 10^5^ cells/well onto the inserts (6.5 mm in diameter with an 8.0 μm pore size) in the upper part of a Costar transwell system (Corning Inc., Kennebunk, ME, USA) and maintained for 24 h at 37 °C in the incubator. The bottom wells were supplied with a solution containing LPS with or without Aze at the indicated concentrations. The effect of Aze on LPS-activated cell mobility was assessed as described in the previous reports [34,35]. Briefly, the treated cells were fixed with 4% paraformaldehyde for 20 min prior to permeabilization with methanol for 10 min and stained with 0.5% crystal violet for 10 min. After gentle removal of non-migrated cells with a cotton swab, the migrated cells were photographed under a Nikon phase-contrast microscope (Nikon Instruments Inc., Melville, NY, USA) and counted in four randomly selected fields in each well. Cell migration was expressed as a percentage of the vehicle-treated control cells.

#### 4.8.2. Wound Healing Assay

BV2 cells were plated onto 24-well plates at a density of 5 × 10^5^ cells/well and incubated at 37 °C until the cells reached 80–90% confluence. The cells were then wounded with a sterile scratcher (SPL, Korea) and treated with Aze at the concentrations of 1, 3, and 10 μM in the presence or absence of LPS, as previously reported [34,35]. The effect of Aze on LPS-induced cell migration was determined by measuring the relative change in the width of the wounds over 24 h using ImageJ software. Images at 0 and 24 h were captured under a Nikon phase-contrast microscope (Nikon Instruments Inc., Melville, NY, USA). The degree of cell migration was presented as a percentage of the vehicle-treated control cells.

### 4.9. Immunocytochemistry

To verify the inhibition of LPS-induced NF-κB nuclear translocation by Aze, immunocytochemistry was conducted following the procedures reported previously [34,35]. In brief, BV2 cells were cultured at a density of 2.5 × 10^4^ cells/well on coverslips placed on the wells of 24-well plates. After incubation at 37 °C for 24 h, the cells were then treated with Aze (10 μM) with or without LPS in serum-free media. Subsequently, the cells were fixed with 4% paraformaldehyde for 15 min, followed by permeabilization with 1% Triton X-100 for 5 min prior to blocking with 5% goat serum for 30 min. Afterward, the cells were incubated with the anti-NF-κB p65 antibody (1:250 dilution) in the blocking solution at 4 °C overnight. The cells were incubated with Alexa Fluor 488-conjugated secondary antibody (1:400 dilution) in the dark for 1 h at room temperature. The coverslips were gently rinsed with PBS and mounted with ProLong Gold Antifade Reagent with DAPI onto microscope slides. The final samples were analyzed using a confocal microscope (Nikon Instruments Inc., Melville, NY, USA).

### 4.10. Statistical Analyses

All data were displayed as the means ± SEM from at least three independent experiments. Statistical significance was analyzed by one-way analysis of variance (ANOVA) using Sigma Plot 12.5 software (Systat Software Inc., San Jose, CA, USA). A *p*-value < 0.05 was considered statistically significant.

## 5. Conclusions

The FDA-approved drug Aze was found to directly bind to the ATP-binding site of JNK3 and exhibited inhibitory effects on JNK3 activity, as indicated by our findings from SBVS and NMR spectroscopy. In addition, cell-based experiments illustrated that this drug suppressed the phosphorylation of JNK and subsequently blocked the phosphorylation of its downstream transcription factor c-Jun in LPS-treated BV2 cells. The activation of JNKs is a critical player in immune response, which is associated with the pathogenesis of many neurological and immunological disorders. We found that Aze inhibited the LPS-induced production of inflammatory mediators, including IL-6, TNF-α, and NO, and cell migration. Furthermore, Aze decreased the LPS-induced nuclear translocation of NF-κB through inhibition of IκBα phosphorylation. Collectively, our data demonstrate that Aze suppresses the LPS-induced inflammation and cell migration through inhibition of the JNK/NF-κB pathway in BV2 cells. This is the first report elucidating the JNK/NF-κB-mediated anti-inflammatory and anti-migratory effects of Aze. Our findings suggest that Aze is a potential candidate for drug repurposing to alleviate the neuroinflammation associated with many neurodegenerative disorders, including AD and PD.

## Supplementary Materials

Supplementary Materials can be found at https://www.mdpi.com/article/10.3390/ijms22169061/s1.

## Abbreviations

AMPPNP	Adenylyl-imidodiphosphate
Aze	Azelastine
COX2	Cyclooxygenase-2
FDA	U.S. Food and Drug Administration
JNK	c-Jun N-terminal kinases
IκBs	Inhibitors of kappa
IL	Interleukin
iNOS	Inducible nitric oxide synthase
LPS	Lipopolysaccharide
NF-κB	Nuclear factor-kappa B
NMR	Nuclear magnetic resonance
NO	Nitric oxide
PDB	Protein data bank
SBVS	Structure-based virtual screening
SDF	Structure data file
STD	Saturation transfer difference
TNF-α	Tumor necrosis factor-alpha
