# Supplementary material for: Suppression of LPS-Induced Inflammation and Cell Migration by Azelastine through Inhibition of JNK/NF-κB Pathway in BV2 Microglial Cells
Source: Int J Mol Sci. 2021 Aug 23;22(16):9061. doi: 10.3390/ijms22169061 (PMC8396433; doi:10.3390/ijms22169061)
Supplement: Supplementary file 1 [file ijms-22-09061-s001.zip › ijms-1339480-supplementary.pdf]

International Journal of *Molecular Sciences*

MDPI

Supplementary materials

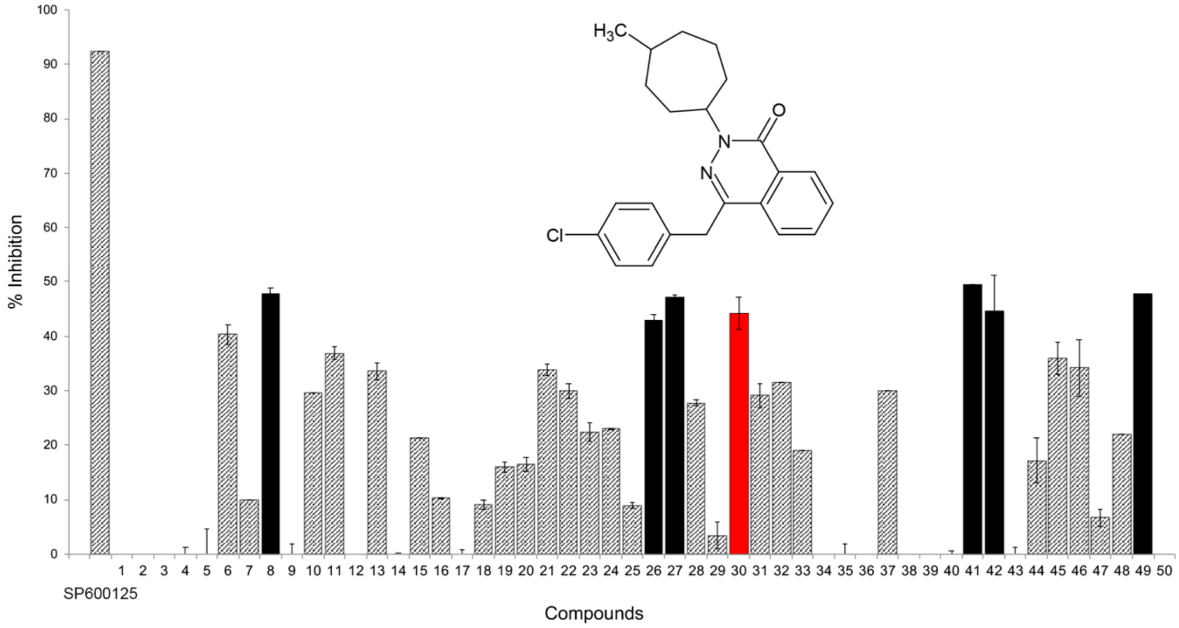

Figure S1. Percent inhibition of JNK3 activity by the selected 50 compounds. The activity of JNK3 was assessed in the presence of each test compound at the concentration of 10 μM, as described in the Materials and methods. Seven compounds exhibited more than 40% inhibition of JNK3 activity. The effect of Aze (Compound No. 30) is highlighted in red, while the effects of the remaining six compounds are presented using filled bars. The % inhibition of JNK3 activity by SP600125 is also shown. Data are expressed as the mean ± SEM. The structure of Aze is shown on the top of the figure.
